# Hydrogels as Potential Nano-, Micro- and Macro-Scale Systems for Controlled Drug Delivery

**DOI:** 10.3390/ma13010188

**Published:** 2020-01-02

**Authors:** Adam Chyzy, Monika Tomczykowa, Marta E. Plonska-Brzezinska

**Affiliations:** Department of Organic Chemistry, Faculty of Pharmacy with the Division of Laboratory Medicine, Medical University of Bialystok, Mickiewicza 2A, 15-222 Bialystok, Poland; adam.chyzy24@gmail.com (A.C.); monika.tomczyk@umb.edu.pl (M.T.)

**Keywords:** hydrogel, drug delivery, polymer, biocompatibility, immobilization of drug

## Abstract

This review is an extensive evaluation and essential analysis of the design and formation of hydrogels (HGs) for drug delivery. We review the fundamental principles of HGs (their chemical structures, physicochemical properties, synthesis routes, different types, etc.) that influence their biological properties and medical and pharmaceutical applications. Strategies for fabricating HGs with different diameters (macro, micro, and nano) are also presented. The size of biocompatible HG materials determines their potential uses in medicine as drug carriers. Additionally, novel drug delivery methods for enhancing treatment are discussed. A critical review is performed based on the latest literature reports.

## 1. Introduction

Drug delivery systems grounded on hydrogels (HGs) are interesting because of their high biocompatibility and biodegradability. These properties are especially relevant for materials used for biomedical engineering applications, an example of which may be drug delivery or tissue engineering [1,2,3]. HGs are water-swollen polymeric networks containing chemical or physical cross-links able to absorb large quantities of water or biological fluids [4]. HGs have a variety of structures, architectures, sizes (from centimetres to sub-nanometres), and functions, and together with other properties, these variables determine HG use for drug delivery [1,2,3,5].

HGs can be prepared from one polymer (homo-polymeric HG), two or more polymers (multi-polymeric HG); they may also contain other nanostructures/nanoparticles in a polymeric network [6,7,8,9]. These polymeric networks can be chemically and physically modified imparting new and unusual properties [9,10,11]. Chemical structures, compositions, biodegradability, biological functions and different physicochemical properties (e.g., mechanical, rheological, spectral, thermosensitive, pH stability) can be modified [2,6,9,12]. These variations influence the performances of HGs and affect loading and releasing properties for drugs [7,9,11,12,13,14,15].

HGs can be used to form microparticles, nanoparticles, micelles and films [15,16]. For HG particles, the particle size (macro, micro and nano) determines the route by which HGs can be delivered into the human body [17,18,19,20,21]. For micro- or nano-sized HGs, the effects of various physical and chemical factors on drug release should be considered [15,21]. Therefore, drug immobilization in a polymer matrix should be considered in the context of controlled release at target sites. Various in vivo and in vitro drug application techniques have been developed with various therapeutic properties [12,22,23,24], including antifungal [25,26,27], antibacterial [28,29,30,31,32], antitumor [33,34,35,36], anti-inflammatory [37,38], immunomodulatory [39,40,41], anti-glycemic [42], antioxidant [32,37,43], tissue repair and regeneration [14,16,44].

The objective of this review is to explore the potential use of HG particles in drug delivery systems with respect to their size (macro, micro and nano). This review also attempts to identify the effects of HG particle size and physicochemical properties on biological performance and medical applications. Finally, novel drug delivery methods for enhancing treatments are discussed.

## 2. Types of Hydrogels

A gel is a liquid treated with gelling substances, including natural polymers (e.g., agar, alginates, and dextran), semi-synthetic polymers (cellulose derivatives) or synthetic polymers (acrylic and methacrylic acid derivatives) [45]. Lipophilic gels (oleogels) are obtained using oil as the dispersing phase. Hydrophilic gels (hydrogels) are obtained using water as the dispersing phase [46,47,48]. Due to their similarity to living human tissues, HGs with controlled drug release are widely used in pharmaceuticals. By modifying their compositions and physicochemical properties (e.g., to impart hydrophilic or hydrophobic character), HGs can be used as drug carriers for external or internal use [49].

HGs are classified using different criteria [50]. The simplest criterion is origin, i.e., natural or synthetic [50,51]. Natural HGs are biocompatible, biodegradable [1] and support cell activity. However, natural HGs have low mechanical strength and large inter-batch variety. Proteins such as collagen or polysaccharides (e.g., chitosan, dextran, and alginate) are examples of natural HGs [52,53,54,55]. Synthetic HG polymers are prepared from polymerizable monomers, including vinyl acetate, acrylamide, ethylene glycol and lactic acid (made from plants, mostly from corn and sugarcane) [56,57,58,59]. Synthetic HGs can be precisely controlled and tailored to achieve desired properties. However, synthetic HGs typically lack bioactivity and have low biodegradability. Hybrid HGs consist of chemically, functionally and morphologically different units [60,61,62,63,64,65]. Biologically active proteins, peptides, nano/microstructures are constituent parts of hybrid HGs and are connected with each other by physical or chemical forces [60,61,62,63,64,65,66]. Because of their construction, hybrid HGs derive their bioactivity from natural materials; furthermore, the easy control over physical and chemical properties of hybrid HGs are due to synthetic material properties [66].

### 2.1. Physical and Chemical Hydrogels

HGs can be classified into two groups based on the type of interactions involved in the creation of the network structure. The first group includes chemical solids [47,51,67,68], wherein HGs form three-dimensional (3D) networks with polymer chains are connected by permanent covalent bonds via cross-linking reactions [69]. Characteristic features of chemical HGs include their ability to swell resulting from interactions among the polymer network, water and the density of connections between polymer chains. Chemical HGs are not homogeneous due to the hydrophobic aggregation of cross-linking agents and high cross-link-density clusters [60].

The second group includes physical (reversible) HGs [51,67,68]. These HGs have chains connected by weak hydrogen bonds, ionic bonds and dipolar or hydrophobic interactions excluding those that dissolve before use [68]. These forces result in non-homogeneous HGs [60]. Examples of physical HG are solutions of agar, gelatine, and polyvinyl alcohol [70,71].

These two types of HGs encompass a wide variety of macromolecular structures formed from cross-linked and entangled linear homopolymers and linear, block or graft copolymers [72,73,74]. The HG networks can be stabilized by reactions of monovalent and polyvalent ions, multiple monovalent ions or complexes containing hydrogen bonds. The properties and applications of these HGs are closely related to cross-linking density, which determines swelling behaviour and the combined properties of solid and liquid phases [51,68,75].

### 2.2. Conventional and Stimuli-Responsive Hydrogels

**Figure 1:**
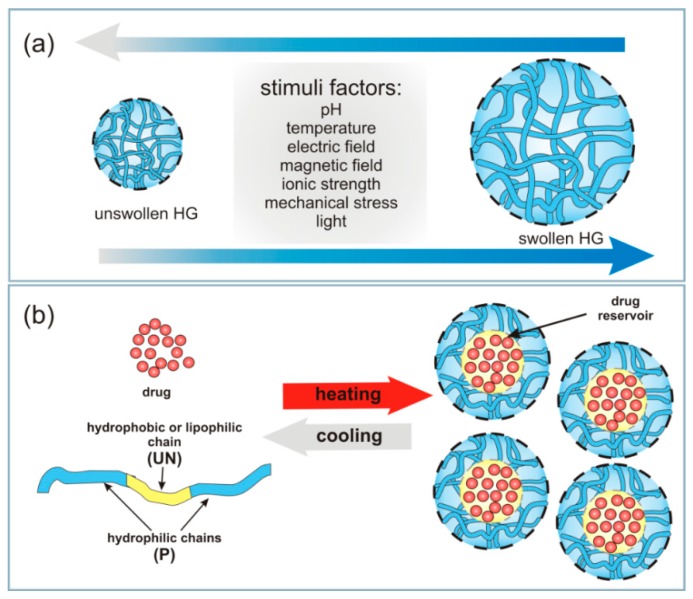
(**a**) Types of stimuli causing HG swelling. (**b**) Schematic illustration of a thermo-reactive HG formation loaded with a drug that uses temperature as a stimulus.

HGs are also classified as conventional and stimuli-responsive. Conventional HGs comprise loosely connected hydrophilic, mostly non-ionic polymers with significant degrees of swelling in water without dissolution [76]. Stimuli-responsive HGs respond to various factors, such as small changes in temperature, ionic strength, pH, electric field, mechanical stress, light and selected substances (Figure 1a) [49,51,77,78,79,80]. Stimuli-responsive HGs can be tailored to react to various types of stimuli in the body, including ionic strength, pH and temperature, to act as potential drug carriers [49,51,77].

#### 2.2.1. Thermosensitive Hydrogels

Thermosensitive HGs have a specific balance between a hydrophilic polymer gel and water molecules. In these systems, even small changes in temperature can disturb this balance and cause a phase change from sol to gel [50]. Such phase transitions often occur when the temperature changes from room temperature to physiological temperature of the human body [46]. This is because temperature influences interactions among water molecules and the hydrophilic and hydrophobic segments of the HG polymer [81].

HGs comprise hydrophilic (P) and hydrophobic (UN) segments arranged in the sequence: P-UN-P or UN-P-UN (Figure 1b). The hydrophilic segments used in HG formulations are characterized by high swelling properties, water solubility and biocompatibility [46]. The hydrophobic segments increase the loading capacity of other hydrophobic molecules, such as drugs, due to the micellization of nanoparticles (Figure 1b) [6,82].

For the proper design of thermosensitive HGs, the phase transition temperature should be identified [83]. The temperature at which the polymer solution separates into two phases is called the critical solution temperature (CST) [46] and is used to classify thermosensitive HGs as positive or negative temperature responsive systems [84,85]. The CST is typically lower for HG polymers with higher molecular weight or hydrogen-bonding interactions. Thermo-responsive HG polymers with lower CST (LCST) remain liquid at low temperature and are called negative temperature HGs. At temperatures lower than the LCST, these HGs swell [86]. When the temperature increases these HGs undergo a sol-gel transition and become insoluble upon heating. The opposite phenomenon is observed for HG polymers with upper CST (UCST, positive temperature HGs), which become soluble upon heating [46]. At temperatures lower than the UCST, positive temperature HGs dehydrate [87]. LCST HGs are used in the medical field because they form gels in situ at physiological temperatures, i.e., 30–37 °C (Table 1) [88]. Examples of synthetic polymers used to develop LCST HGs include copolymer blocks of poly(ethylene oxide) and poly(pentapeptide), poly(*N*-isopropylacrylamide) (PNIPAM), and poly(*N*,*N*-diethyl acrylamide) (PDEAM) [88]. Natural LCST polymers include cellulose derivatives, chitosan and gellan gum (Table 1) [88,89,90].

#### 2.2.2. Photo-Responsive Hydrogels

Light-responsive HGs are subjected to sol-gel changes under the influence of light at different wavelengths: infrared (IR), ultraviolet (UV), or visible (VIS) [50]. The initiation of sol-gel phase transitions in photo-responsive HGs using light is straightforward and non-invasive making these HGs a potential source of functional material in biomedicine, e.g., for drug delivery [50,97,98]. To prepare a photo-responsive HG, a light-responsive chromophore group is attached to HG polymers [99,100]. Incident light initiates swelling/deswelling processes or sol-gel phase transition to release drugs at desired locations. UV-light-responsive HGs undergo photopolymerization or photocleavage depending on the exposure length [101,102]. Light wavelength is also extremely important because it affects the drug release control process [103].

#### 2.2.3. pH-, Electric- and Magnetic-Responsive Hydrogels

pH-responsive HGs have specific pH-dependent physicochemical properties [104]. These HGs have acidic and alkaline groups associated with HG polymer chains [50,105]. The human body is a dynamic environment with different tissues having different pH ranges, which provides delivery means for in vivo pH-responsive HGs in pharmaceutical and biomedical applications [106,107,108].

Ionizable groups embedded in the polymer network of pH-responsive HGs accept or donate protons in response to pH change, resulting in changes to the structure and solubility of the HGs and consequently, swelling or deswelling (Figure 1a). The most frequently used synthetic monomer with acidic characteristics are acrylic acid, methacrylic acid, maleic anhydride, *N*,*N*-dimethylaminoethyl methacrylate and sulphonamide-containing polymers [2,18,20,91,109]. Weak synthetic polybases, such as aromatics 4-vinylpyridine, 2-vinylpyridine, poly(vinyl imidazole), poly(*N*,*N*-dimethyl aminoethyl methacrylate), and poly(*N*,*N*-diethyl aminoethyl methacrylate), accept protons at low pH [50,110,111]. There is also a large group of natural polymers used in pH-responsive HGs that has advantages over synthetic polymers due to their degradability within the human body, making them excellent biocompatible components in drug delivery [9,33,88,112].

There are also HGs that respond to external physical stimuli, like electric and magnetic fields [52,105]. Magnetic or electrically responsive HG networks have been developed for drug-delivery applications. The influence of an electric field causes electro-responsive HGs to swell or de-swell, which can be adapted for drug delivery systems [113]. Similar applications have been determined for magnetic field-based stimuli, especially high frequency fields, which are much safer for humans than electric fields or UV light [114]. Magnetic and electrical stimuli-responsive HGs allow for the study of cell behaviour by altering hydrogel properties in situ. These stimuli-responsive HGs can serve in potential drug delivery systems for targeting and localizing to delivery sites for controlled drug release upon application of electric or magnetic fields [115,116,117].

### 2.3. Other Classifications for Hydrogels

HGs could also be classified by the substrates used for their production. Polymer networks may be synthesized from linking monomers to form high-molecular weight polymers. Hydrophilic monomers, such as polyphenylene oxide (PPO) or polyethylene glycol (PEG), are copolymerised with crosslinkers to form networks for drug delivery [118,119]. When prepolymers (oligomers) are used as synthetic substrates, different types of HGs are obtained. A series of model networks consisting of PEG, tetra-PEG gels, have been prepared by Sakai et al. [119,120]. Small-angle neutron scattering measurements confirmed, that network structure is extremely uniform, even in the presence of different functional groups at the ends, i.e., amine group and succinimidyl ester group [121,122]. Additionally, the modified with ionic liquid form of tetra-PEG gels possesses high ion conductivity and high mechanical properties [123,124]. For example, polyurethane-based HGs are used in the fabrication of flexible medical devices, prosthetics for example [125]. Other substrate-specific HGs are those with network structures made by crosslinking hydrophilic polymer chains, e.g., chitosan crosslinked with glutaraldehyde [75].

Due to large differences in their chemical compositions, HGs are sometimes broadly classified [51,126]. For example, in terms of the types of monomers used to prepare HGs (polymeric composition), three groups of HGs are recognized: (i) homopolymeric HGs (consisting of one type of hydrophilic monomer); (ii) copolymeric HGs (two or more monomer types of which at least one is hydrophilic), and (iii) multipolymer HGs (obtained from more than one type of polymer).

Multipolymer interpenetrating polymeric (IPN) HGs represent a class of HGs made of two independent cross-linked synthetic and/or natural polymers [51,75,127,128,129]. HGs there are two polymer networks, one of which is polymerized around and within the on the second polymer network, and what is very important, there are no covalent linkages between those two polymeric networks.

HGs may take significantly different morphological forms, which can be a criterion for their division depending on the state of aggregation (physical form) [17,105,129]: (i) solid (amorphous, semicrystalline or crystalline); (ii) semisolid (for example shear-thinning HGs), and (iii) liquid [17,51,75,130]. Solid HGs can be useful in formation of functional tissues [75,131]. At room temperature they are solid in nature with strong cross-linked network structure [17]. Solid HGs swell in the presence of water or other hydrophilic solutions like buffers or biological fluids. Semisolid HG consists of two types components where at least one possess biological nature, e.g., plant resins or gums [17]. The shear-thinning HGs also belong to this group [17,132,133]. These HGs can be pre-gelled outside of the body and injected by applying shear stress and flow like low-viscosity fluids. The shear-thinning behaviour is a result of reversible physical cross-links. That group of HGs is often called bio- or muco-adhesive HGs according to their specific adhesive properties [16,75,134]. Liquid HGs are injected in liquid form and a sol-gel transition is performed inside the human body [19,109,135,136,137]. The resulting HGs takes the shape of space available at the injection site which allows to achieve the sol–gel transition with variety of strategies. Slow-gelling systems based on gelation mechanisms (charge interaction, stereocomplexation, click chemistry, etc.), termo- or pH-responsive systems are good representatives of this group.

It is also possible to classify HG based on ionic charges into the following four groups: (i) neutral (no charge, e.g., dextran); (ii) anionic (negative charge) like carrageenan; (iii) cationic (with positive charge) such as chitosan, and (iv) amphiphilic (having the ability to strongly interact with both polar and nonpolar solvent molecules—such as collagen) [17,138,139,140,141,142,143].

## 3. Types of Physical Appearance of Hydrogels

HGs have a number of properties, such as oxygen and nutrient permeability and the ability to bind, transport and control drug delivery and release, attractive for use in biological applications. Different tissues have different requirements and would be better served using different types of HGs [144]. HGs can be formed in almost any shape and size [145], e.g., as a matrix, a film, microparticles, nanoparticles, macro-beads, membranes, or coatings, depending on the polymerization technique [46,51,82,146]. HGs are altered to improve their compatibility with hydrophobic or hydrophilic compounds or drugs with different properties [147,148].

Polymeric HGs are classified as macro-, micro- and nanogels with respect to particle size [17,149]. Microgels, cross-linked structure is large, usually from millimetres to centimetres [150]. A group of gels called microgels, whose particles are smaller, separate and cross-linked, having a size between 1 µm and 100 nm [151]. Nanogels are those gels whose particles have a size smaller than 100 nm [49,106,152]. It should be noted that gels with particle sizes only slightly larger than 100 nm were called quasi-nanogels [153].

Microgel is a submicron- or micron-sized network polymer which is highly swellable but insoluble in water [154]. Cross-linked microgel particles possess special kind of control over particle shape and size, which is probably due to specific extensive swelling pattern [155]. Microgels are obtained in two ways. One of them is molecular assembly of existing polymer molecules in aqueous solutions. The other one, particle-forming polymerization, includes two types of polymerization: precipitation and inverse emulsion polymerization [154,156].

Nanogels are prepared in water by self-aggregation of polymers, mostly of natural origin; natural polysaccharides (dextran, pullulan) or cholesterol-containing polysaccharide [14,157]. The dimensions of these HGs are usually of 20–30 nm. Such a small size causes the use of nanogels for cell targeting, because swelling caused by pH changes in the surrounding environment is the reason to release the entrapped drug [158]. Nanogels react to the external stimuli changes much quicker than macrogels. The reason for this is probably their small size and short relaxation time [159,160]. Swelling and deswelling properties and small size of biocompatible and biodegradable nanogel particles are the cause of easy crossing the blood brain barrier [21,149,153].

Obtaining a specific shape of HG at a nanoscale is difficult. HG nanofibers, for example, can be obtained by the by electrostatic spinning method [161]. HGs are also given the form of microcapsules and microspheres, whose size is 1–1000 µm. This allows more protection of substances enclosed in the interior of microcapsules and microspheres. HGs can form micelles in an aqueous environment, as a result of aggregation of amphiphilically connected blocks or end-modified polymers. It is often difficult to differentiate micelles from reversible HGs, because HGs can form micelles above a appropriate concentration called the micelle gel concentration [162]. Similarly, biodegradable nanoparticles of HGs are classified as nanocapsules or nanospheres having drug molecules adsorbed on the surface or closed inside [163,164].

There also exist the group of in situ-gelling HGs, which undergo a sol-gel transition inside the human body [165]. That group of HG always adapts to the free space with its shape. An example of such HGs are shear-thinning HGs. Because of reversible physical cross-links shear-thinning hydrogels pre-gelled outside of the human body, after shear stress injection can regain its initial stiffness.

Despite of many nano- and microgels advantages, it should remember about the possibility of using macroporous HGs and the benefits resulting from it. Macroporous HGs can mechanically collapse at up to 90% and recover reversibly almost immediately after injection in the human body. Such macroporous HGs allow for the preparation of highly defined shapes for drug delivery [151].

## 4. Immobilization of Drugs in Hydrogel

### 4.1. HG Structure and Physical Properties

A physical and chemical properties, including structural parameters, swelling behaviour, diffusive characteristics, and surface properties, determine the biomedical and pharmaceutical applications of HGs [5]. After the creation of porous HG structures, different drug molecules may be incorporated into the scaffolding (post-loading) or in the network during formation (in situ loading); these drugs are subsequently released from the hydrophilic matrix to biological media. Thus, network mesh size (or pore size) is a major parameter which determines the sizes of the drug system that can be immobilized in the porous matrix of the HG. The network mesh size (*ξ*) indicates the distance between adjacent junctions, cross-links and tie points. Cross-links may be chemical (mainly covalent bonds) or physical (e.g., electrostatic, hydrophobic and dipole-dipole) [5].

**Figure 2:**
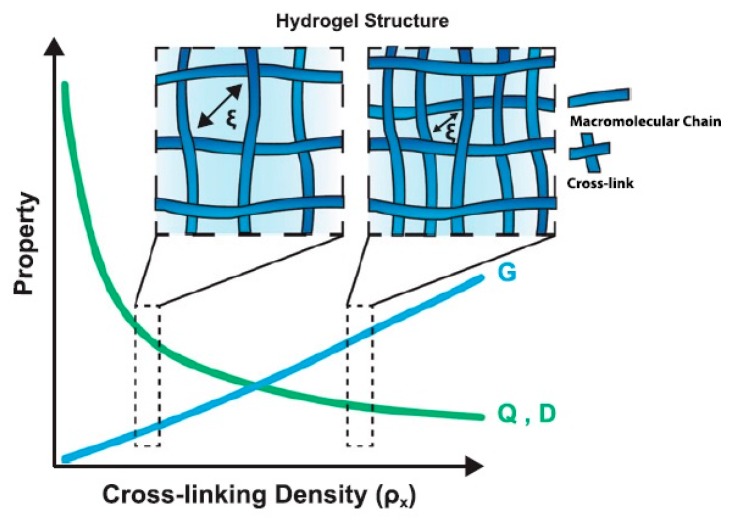
Relationship between the cross-linking density and HG properties. Two network structures representative of low and high cross-linking densities are depicted to demonstrate the relationship between the cross-linking density and basic HG properties of highly swollen, non-ionic gels, including shear modulus (*G*), equilibrium volumetric swelling ratio (*Q*) and diffusivity (*D*). As the cross-linking density increases, the mesh size (*ξ*) decreases, which is a measure of the space available between macromolecular chains for the diffusion. Reprinted with permission from Ref. [5].

The cross-linking density (*ρ*_x_) of HGs, which refers to the number of cross-links in a given volume, is a parameter that defines the structural properties of HGs [5,166,167]. Relationships between cross-linking density and HG properties (e.g., shear modulus, equilibrium swelling ratio, drug diffusivity) are presented in Figure 2 [5]. These parameters allow for the prediction of HG release behaviour.

From experimentally measured volumetric swelling ratios of HGs (*Q*), the average molecular weight can be calculated between cross-links (${\bar{M}}_{c}$) and the equilibrium polymer volume fraction (*v_2_*) of a given network [167]. There are many equations describing the relationship between the network structure and its physical properties, which allow the calculation and prediction of transport properties of homogenous porous materials:

(1) $$\xi =\;v_{2}^{-\frac{1}{3}}\;{\left(\bar{r_{0}^{2}}\right)}^{\frac{1}{2}}\;$$

(2) $$\;\frac{D_{g}}{D_{0}}=\;\left(1-\;\frac{r_{s}}{\xi }\right)\exp\left(-Y\left(\frac{v_{2}}{1-v_{2}}\right)\right)\;\;$$

(3) $$\;Q\;\sim \;{\left(\bar{M_{c}}\right)}^{3/5}\;$$

where *ξ* is the mesh size; ${\left(\bar{r_{0}^{2}}\right)}^{\frac{1}{2}}$ describes the root-mean-squared end-to-end distance of the polymer chains in the unperturbated state; *D_g_* means the solute diffusivity in e swollen gel; *D_0_* means the diffusivity of the solute in the swelling solvent; *r_s_* is a sign of the radius of the solute; *Y* is the proportion of the critical volume needed for a successful translational motion of the solute molecule to a medium free volume attributable per molecule of the liquid [167,168,169]. After some simplification, these equations can be used to find the correlation between all mentioned parameters (Equation (4)) for highly swollen, non-ionic HGs, and scale with ${\bar{M}}_{c}$:

(4) $$1-\;\frac{D_{g}}{D_{0}}=\;\frac{r_{s}}{\xi }\;\sim \;{\left(\bar{M_{c}}\right)}^{-\frac{7}{10}}$$

The HG network structure is also important in determining the mechanical properties of porous material. The network structure controls swelling behaviour and drug release in biological environment. Swelling behaviour is defined as the ratio of the volume of the water-swollen gel to the volume of dry polymer and is indicative of the water content of the swollen HG. However, higher water content resulting from high network porosity is more beneficial for immobilizing active substances and can simultaneously lead to polymer degradation and prevent the controlled delivery of drugs. In this context, the cross-linking density must be high enough to allow for the immobilization of the active substance in the polymer matrix. Beyond the structural properties, the chemical composition of HGs regulates the final biochemical properties, e.g., charge, hydrophilicity and bioactivity.

**Figure 3:**
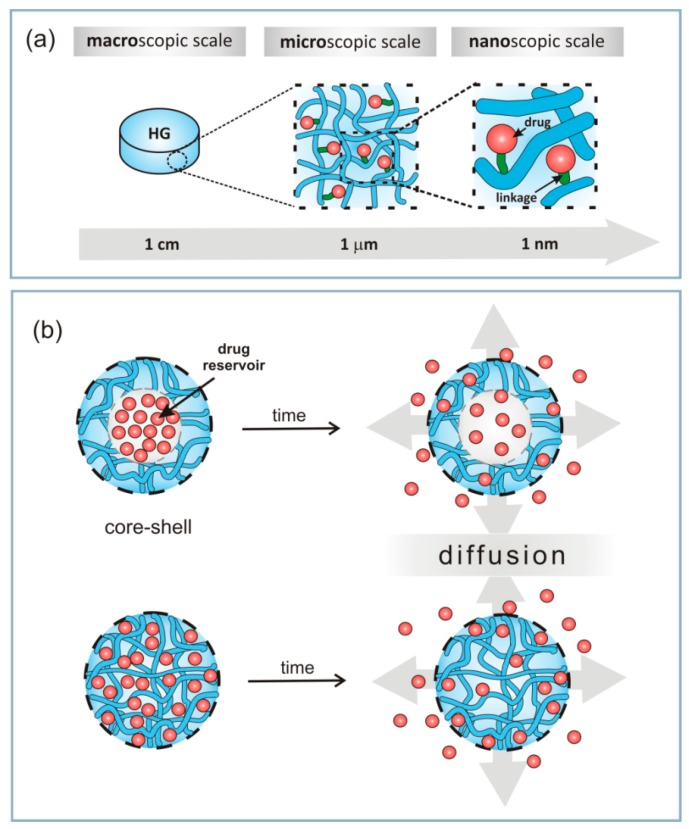
(**a**) Macroscopic design of HGs including the size and porous structure. (**b**) Schematic structures of micro- and nanogels showing the release of drugs.

In this brief discussion, we highlighted the structural properties of macroscale HGs. These HGs have consistent physicochemical properties throughout, and drugs can be distributed homogeneously in “bulk HGs” (Figure 3a). Macroscopic HGs have dimensions of millimetres to centimetres and are typically surgically implanted or arranged in contact with the patient’s body to achieve transepithelial drug delivery [17,137].

**Figure 4:**
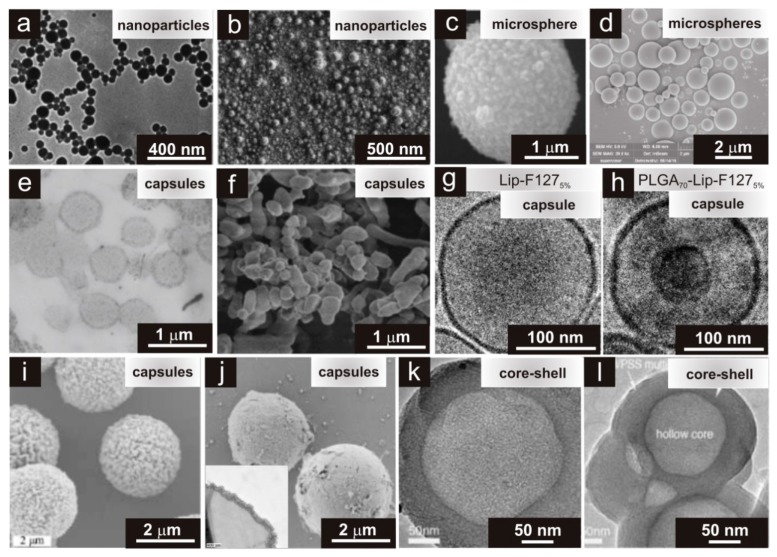
Morphology (structure) of HGs observed by (**a**,**c**,**e**,**g**,**h**) TEM and (**b**,**d**,**f,i,j**) SEM at different magnifications (the scale bars are marked on the images) obtained by post-loading or in situ loading drugs. (**a**,**b**) Piperlongumine-loaded poly(ethylene glycol)-poly(dehydroabietic ethyl methacrylate) HG nanoparticles [172]. (**c**) Silver alginate HG microspheres [173]. (**d**) Thermosensitive HG microspheres loaded with tenofovir [174]. (**e**,**f**) Polyacrylamide-poly(*N*-isopropylacrylamide) HG capsules loaded with doxorubicin hydrochloride [175]. (**g**) Unloaded lipid capsule (Lip) with F127_5%_ (5 wt% content of Pluronic F127) [176]. (**h**) Doxorubicin-loaded poly(lactic-co-glycolic acid) (PLGA) core-lipid shell (Lip) with F127_5%_ structure [176]. Smart organic/inorganic HG polyelectrolyte capsules (poly(allylamine hydrochloride): (**i**) hollow and (**j**) hydroxyapatite [177]. (**k**,**l**) Core-shell mesoporous silica spheres (**l**) loaded with ibuprofen [178]. Reprinted with permission from Refs. [172,173,174,175,176,177,178]. The Royal Society of Chemistry, Hindawi, Nature Group, Springer and Wiley-VCH Verlag GmbH & Co.

Other structures, e.g., micro- and nanogels, draw attention to their use as injection materials (Figure 3 and Figure 4). Their relative scale (1 µm to 100 nm microgels and 10–100 nm nanogels) determines the course by which HGs can be delivered to the human body [17,18,19,20]. Small HG particles are needle-injectable, provide large surfaces for bioconjugation and enhance penetration through tissue barriers [135,136,170,171].

Drug delivery based on micro- and nanogels are superior when compared with macrosystems (Figure 3b) because: (i) the smaller sizes of these HGs allows for active and passive drug targeting; (ii) controlled drug release leads to improved therapeutic impact and reduced side effects; (iii) drug loading takes place with no chemical reaction (environmental stimuli); (iv) the smaller HGs may infiltrate into tissues via paracellular and transcellular routes; and (v) the smaller HGs are biocompatible and biodegradable [15,179]. Examples of different structures/morphology of HGs are presented in Figure 4. These systems were observed using TEM (transmission electron microscopy) or SEM (scanning electron microscopy). We can distinguish two types of HGs: (i) “core-shell” systems and (ii) homogenously dispersed drugs (Figure 3b and Figure 4). In the core-shell system, a shell is formed by HGs around a central core is drug reservoir (Figure 4e–h,l,k). This structure can be produced in the form of capsules, spheres or slabs, having a high concentration of the drug in the core of the systems, which facilitate the constant release of the drug in the body [126,174,175,180,181]. In the second system, the drug is dispersed homogeneously in a polymer matrix (Figure 4a–d), which in contact with a bio-fluid usually begins swelling, and takes forms of nanoparticles, polymeric micelles, microspheres, etc. [68,172,180,181]. For both types of HGs, releasing of drugs is time-depended process and it is mainly diffusion controlled. Drug releasing from the HG network will be discussed in detail in the next paragraph.

### 4.2. Immobilization of Drugs

HGs due to their 3D structures may be applied as carriers for drugs, proteins, lipids or cells [15,17,68,182,183]. Additionally, a large amount of water within their structures makes a convenient environment for the immobilization of drugs. However, 3D structures of HG allow the immobilization of drugs in their matrix, in this regard, when immobilization process of active substances is performed, two aspects must be taken into account. The large pore size of HG (large mesh size), as well as the high water content causes that water-soluble drugs with small particles quickly escape from the network, thanks to which they have a short release time [182]. Drug release is much slower when the drug particle size is comparable to the mesh size [17]. When the drug particle size exceeds the mesh size, then the drug is physically trapped in the network [17]. To minimize these problems, the pore size in HG (mesh size) should be “matched” to the size of the immobilized drug.

**Figure 5:**
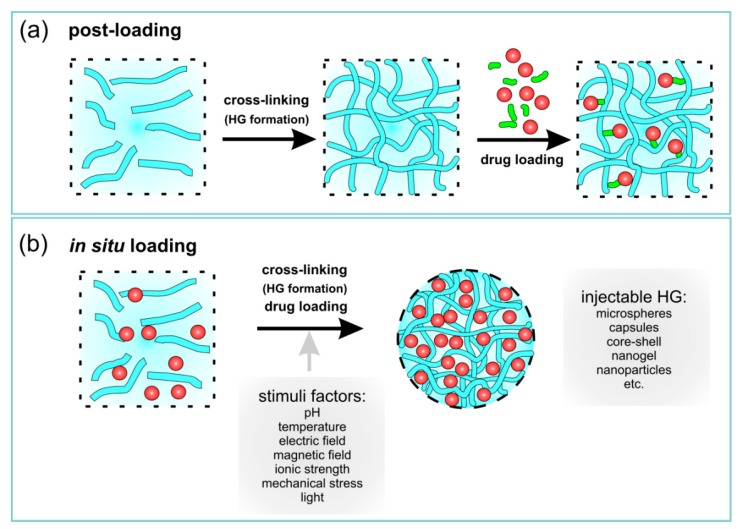
Schematic representation of drug loading in a HG network using (**a**) post-loading and (**b**) in situ loading mechanisms.

There are many methods leading to loading of drugs in HG networks/spheres/capsules, and the choice of method depends mainly on the place where the drug should be delivered. Briefly, drugs can be loaded into HG matrices in two ways: post-loading and in situ loading (Table 2 and Figure 5). HGs have proven to be very efficient for local drug delivery, which could lead to prolonged and faster release. Some examples of HG applications are presented in Table 2; different structures are presented in Figure 5.

Briefly, the post-loading drug process consists of forming a polymer HG matrix (a preformed HG) and immobilizing the drug in the polymeric network (Figure 5a). HGs swell in drug solutions till equilibrium. Depending on the HG macro- or microscopic size, HGs are administered by more or less invasive procedures. Preformed HG gels outside the human body, while being quite simple films or sticky solutions that retain their rheological or mechanical properties after administration [184].

Drugs can be physically or chemically immobilized in HGs and can also be loaded into other species (secondary delivery vehicles) to provide the appropriate environment for targeting. Preformed HGs are solid even when injected [184].

In situ loading of drugs is associated with the simultaneous formation of injectable HGs in the body and the encapsulation of drugs (Figure 5b). Briefly, the whole process shows a sol-to-gel transition in situ that form after injection in vivo.

The resulting HG take the shape of the available space. In situ gel formation usually includes the subsequent steps: (i) gelation as a response to the changes of temperature or pH changes (thermoresponsive or chemical responsive polymers; i.e., “smart” polymers), (ii) ionic or covalent cross-linking, (iii) solvent exchange or crystallization, and/or (iv) thickening upon removal of the injection shear. These external stimuli are shortly summarized in Figure 1a (stimuli factors).

Taking into account the lifetime of HGs, their formation mechanism can be categorized as: (i) pre-gelation (polymer precursor in solution), (ii) therapeutic window (after injection and gelation containing drug), and (iii) degradation (HG degradation products) [18]. These systems may be homopolymeric or multipolymeric or may also be composed of different components, including other systems (e.g., capsule, microgels, and nanoparticles) serving as drug carriers. This latter mentioned gel systems are characterized in the next section.

#### Immobilization of Drugs in Hydrogel Composites

Some systems containing drugs in matrix/capsules are poorly water-soluble or insoluble. In this sense, soluble therapeutic agents are poorly retained in HGs due to their hydrophilic nature. In this case, there are many problems with loading process of drugs due to their tendency to aggregate resulting in high local concentrations causing toxicity [195]. To overcome these problems, HG composites have been created to exploit the hydrophilic-hydrophobic nature of various components [192,196]. HG composites contain polymeric networks (hydrophilic) swollen with water and nanostructures/microstructures with different physico-chemical properties [192,197,198,199]. These composites represent a new class of materials with new properties.

The inclusion of nanomaterials in the HG polymer network is an interesting way to adjust the mechanical properties of HG and/or to provide the composite with sensitivity to external stimuli [192,196,197,199]. Different nanomaterials have been immobilized in polymeric networks, including inorganic nanoparticles [200,201], carbon nanomaterials [202], and lipids [99]. Nanoparticle systems have gained considerable attention by being one of the most interesting and promising biomedical materials with the exceptional physicochemical properties, controlled shapes, nano-sized characteristics, comprehensive modification options and well-defined multi-functionality [83]. The preparation of HG composites may be performed using physical and chemical forces.

**Figure 6:**
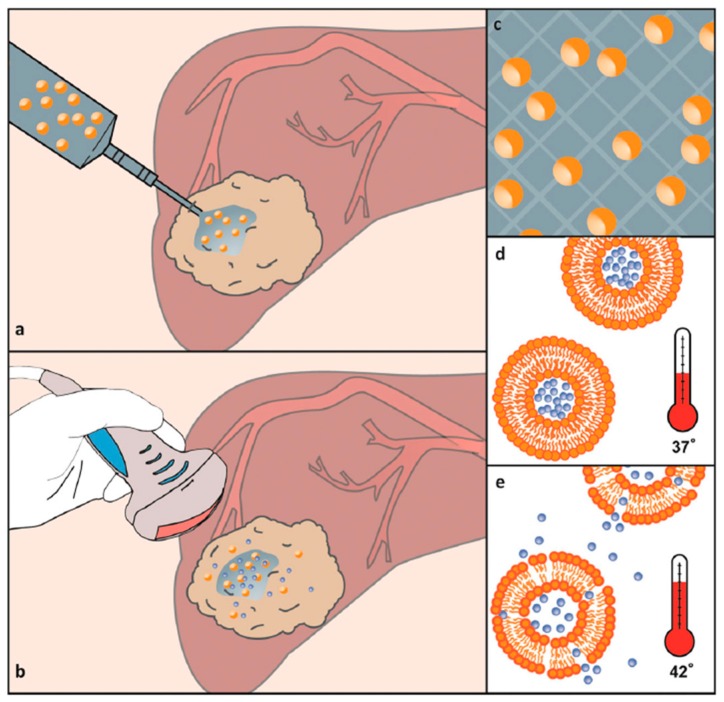
(**a**) Fully syringe-injectable Lipogel consisting of a chitosan/β-GP thermoresponsive gel containing a suspension of DOX-loaded thermosensitive liposomes. (**b**) Release from the gel in situ is controlled using minimally invasive hyperthermia, which is achievable using high intensity-focused ultrasound. A small portion of the drug-loaded liposomes is released from the Lipogel, maximizing the drug delivery distance from the gel implant. (**c**) The majority of liposomes are locked into the gel upon initiation of cross-linking during thermogelation. (**d**,**e**) Liposomes sequester the majority of the drug at body temperature but rapidly become more permeable upon mild hyperthermia and release their drug payload. Reprinted with permission from Ref. [187].

To overcome the incompatibility of hydrophobic drugs and hydrophilic HG networks, lipid nanoparticles (LPNs) are frequently used to promote good solubility. LPNs have been used for dermal, mucosal, transdermal and intramuscular applications [176,182]. Three types of LPNs have been used in drug delivery systems: (i) lipid nano emulsions (LNEs, where the core is composed of liquid lipids), (ii) solid lipid nanoparticles (SLNs, where the core has lipids in a solid state at room and body temperatures), and (iii) nanostructured lipid carriers (NLCs, where the lipid core is a heterogeneous mixture of solid and liquid lipids) [182]. The lipophilic core of LNPs entraps active ingredients, whereas the surfactant membrane (consisting of phospholipids) ensures the stability of LNPs in hydrophilic environments. In pure lipophilic form, these systems have unsuitable rheological properties and therefore, require structural modifications. These modifications result in the formation of LNP-HG composites [187] as shown in Figure 6.

An in situ gelation of chitosan/β-glycerophosphate (GP) and thermoresponsive liposomes was performed [187]. This biocompatible and biodegradable HG was used as a matrix for lysolipid thermally sensitive liposomes (LTSL) loaded with doxorubicin (DOX). LTSLs are bi-layered spherical vesicles that rapidly change structure upon mild hyperthermia (41–43 °C), creating openings in the liposome. DOX was loaded into LTSL by changing solution pH. DOX delivery was also based on the pH-sensitivity of liposomes to acidic pH. A schematic of the controlled-delivery of chitosan/β-GP/DOX-loaded LTSLs and DOX release is presented in Figure 6.

**Figure 7:**
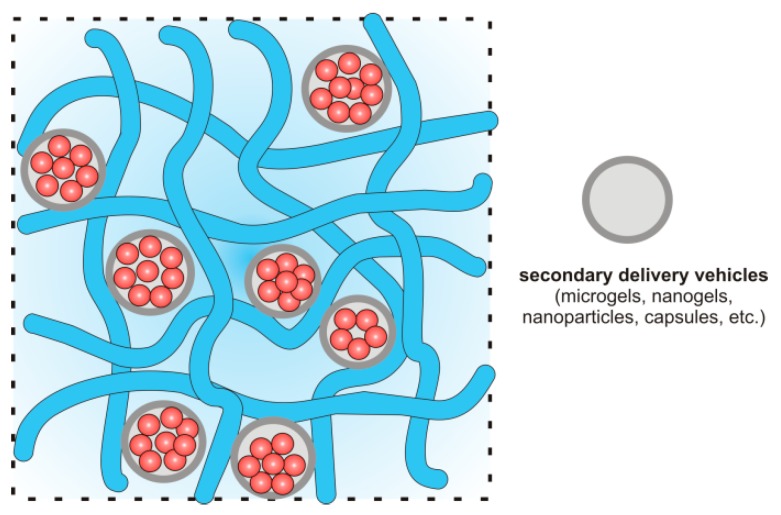
Structure of “plum pudding gels” (PP gels). Composite HG containing a drug embedded in a secondary controlled delivery vehicle (microgels, nanogels, nanoparticles, capsules, etc.).

The next very interesting group of composite HGs are “plum pudding gels” (PP gels), which are schematically shown in Figure 7 [179,187,188,192,194]. This type of the composite HG contains microgels or nanogels inside a bulk HG network, which improves the loading and release of drug. In the PP gel structure, the microgels or nanogels act as reservoirs for drugs and can be incorporated into a conventional macroscale HG at the different concentrations to provide two-component gel matrices.

From a drug delivery perspective, these systems are particularly interesting because the drug delivery limitations of microgels and HGs are minimized, whereas their synergistic effects are observed as soft nanocomposites [179]. The presented chitosan/β-GP/DOX-loaded LTSL system belongs to this group of composites [187]. Other similar systems are listed in Table 2.

### 4.3. Release Mechanism of Drug from Hydrogel Matrices

**Figure 8:**
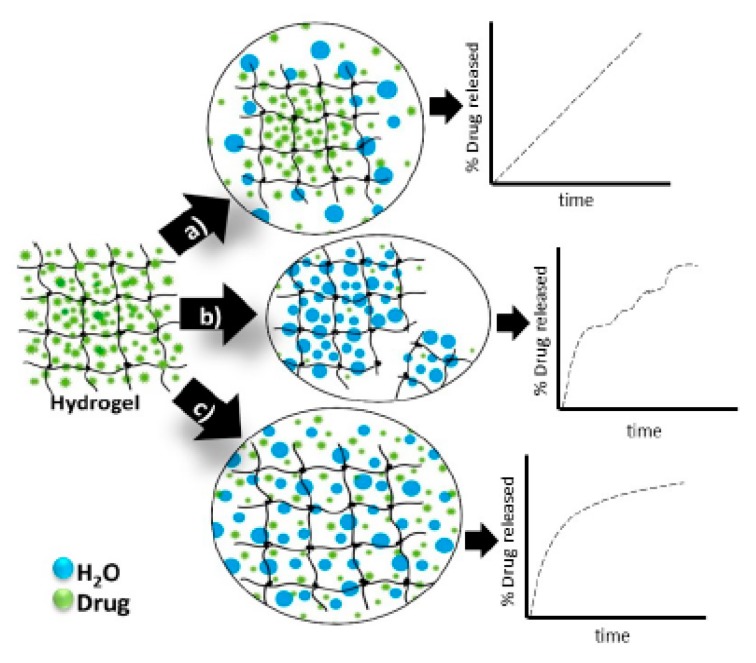
Hydrogels drug release mechanisms and their respective kinetic profiles: (**a**) drug diffusion, (**b**) degradation of the polymeric matrix and (**c**) swelling. Reprinted with permission from Ref. [203].

The physical and chemical properties of HGs affect their delivery properties, including controlled release. HG structure, diameter, and cross-linking agent density affect the rate of drug diffusion. The kinetics of drug release from HGs is connected to the chemical structure and crosslinking density of the materials, including the HG matrix monomers and coating (Figure 8) [203].

The use of HG networks increases the local concentrations of pharmaceuticals and their slow release at delivery sites. Suitable controlled-release mechanisms include (Figure 8): diffusion [17], swelling [68], chemical and environmental stimuli (degradation or deformation) [67]. Drug release from HGs in response to environmental stimuli may occur due to changes in pH [110,204], temperature [110,205,206,207], electric field [109,111,208] or ionic strength [209,210]. Some ideas are schematically presented in Figure 8 [17].

## 5. The Use of Hydrogels in Modern Pharmacy

Interest in using HGs has not decreased over the years. The development of specially modified HGs has provided new pathways for drug delivery and offers advantages as a vehicle for active substances [191,192,211,212,213]. One of the most important advantages is administration route versatility. HGs offer several routes: oral, injection (both intramuscular, into bone and subcutaneous), rectal, vaginal, through wounds, and ophthalmic (Table 3). Drug delivery is strongly connected to drug molecule size and is dependent on administration route. As mentioned previously, HG delivery systems can be classified into three main categories based on their size: macro-, micro- and nanosized.

We present some examples of HGs applications in drug delivery. Most informative reports from the last couple of years regarding HGs in health care have been well summarized; however, the literature on HGs is constantly increasing, as well as interest in HG materials. Herein, we highlight only a few studies that only begins to hint at the wide applications of HGs (Table 3).

### 5.1. Application of Hydrogels for Oral Administration

Oral administrative routes are classic and accepted means for delivering drugs. The vast majority of medicines available on the market are taken through oral routes. Several pills are often taken daily at appropriate intervals to achieve effective therapy. This reduces associated risks of skipping or missing doses and thereby lowering the effectiveness of the therapy. The development of delayed release capsules that prolong the delivery of active substances has been a breakthrough and has allowed for increased compliance. With these delayed release capsules, the amount of tablets consumed is reduced, usually to only one per day. Acid-sensitive drugs require protection against harmful effects of gastric juice typically encountered when delivering drugs vial oral routes. Protection can be offered by using special tablets coated with polymers soluble only at basic pH, such as in the intestines. As a result, drugs survive transit through the stomach and are released only in the intestines thereby promoting higher absorption into the bloodstream.

HGs have also been explored for oral delivery applications. The appropriate selection of gel or the addition of pH-dependent coatings enable the controlled release of active substances from HGs.

The first modification was presented by Panahi et al. [214] (Table 3). Chitosan (CTS)-based gels with acrylic acid (AA), acrylamide (AAm) and polyvinylpyrrolidone (PVP) were prepared [214]. During HG formation, a mineral (montmorillonite, MMT), which has ability to absorb water, was used. Clarithromycin (CAM), a macrolide antibiotic, was used to eradicate *H. Pylori* from the gastrointestinal tract was immobilized in the HG network. It was important to achieve a prolonged release of the antibiotic to increase the chances of an effective therapy. MMT increased the pore diameter in the gel structure, which increased the immobilization of the active substance. However, this also prevented solvent from readily reaching CAM through more intricate pathways in the network, which complicated drug release.

The dependence of the release of the drug on the pH level was explored by Qi et al. [215] (Table 3). An HG system was prepared based on salecan (beta glucan) with pH-sensitive poly(2-acrylamido-2-methyl-1-propanesulfonic acid) (PAMPS). The system had the ability to take or donate protons depending on the pH of the environment, while reducing or increasing the network volume. The authors showed that salecan in combination with PAMPS had the ability to release the active substance depending on the pH of the environment. Using insulin as an exemplary drug, at acidic pH insulin was released at a lower level than at neutral or slightly alkaline pH.

Insulin release from HGs was more extensively studied [216]. Special nanocarriers based on methacrylic acid were synthesized. In a neutral environment, the methacrylic polymer chains started to repel each other, thereby loosening the nanocarrier network and allowing the release of insulin. This property was exploited in preparing HA-doped HG. In the intestines, where the pH is above 7, insulin release increased dramatically compared with release in the stomach. By using HGs as a drug carrier, the release of insulin was extended over time.

In another study, a unique biodegradable, super porous, swellable and pH-sensitive nanocellulose reinforced CTS HG was prepared for the oral administration of curcumin [217]. The in vitro degradation of HG was dependent on the swelling ratio and the number of cellulose nanocrystals (CNCs) in the HG. All HGs showed maximum swelling ratios greater than 300%. The drug release occurred in simulated gastric media; the drug maintained its chemical activity after in vitro release. According to this study, CNC-reinforced CTS HGs can be used to improve the bioavailability of curcumin for absorption from the stomach and upper intestinal tract.

Finally, tetrakis(hydroxymethyl)phosphonium chloride was used as a crosslinking agent in a Mannich reaction to obtain chitosan-based HGs [218]. These pH-sensitive HGs showed low toxicity, high biocompatibility, and allowed for the modified release of encapsulated drugs, namely camptothecin, for 48 h. According to the obtained results, the oral administration of camptothecin through HGs provided low concentrations of the drug at the absorption site, avoiding carrier saturation and reducing intestinal toxicity [218].

### 5.2. Hydrogels for Dermal Applications

Due to their fairly compact consistency, HGs can also be applied to wounds or other skin issues. Commercially available HG-based dressings used for exudative wounds, pressure sores or burns, exploit the adhesive properties and ability of HGs to absorb liquids. The addition of antibacterial substances may further improve the applications of HG dressings.

Researchers from Southwest University in China developed a sericin (SS) and polyvinyl alcohol (PVA)-based HG (Table 3) [219]. This gel showed good biocompatibility, humidity and self-healing properties, i.e., ideal for dressings. Gentamycin sulphate, a known antibiotic substance and aminoglycoside, was added to this HG. To determine the properties of the obtained antibacterial HG, a number of in vitro tests were carried out, which measured wettability, swelling, microbiological activity, drug substance release, cytotoxicity, and immunotoxicity. The studies showed that the antibacterial HG ensured a prolonged release. For a deeper analysis of the HG properties, a model infected tissue was prepared. Tests confirmed that the HG was cytocompatible with mammalian cells and did not affect the growth of healthy cells.

The material used in wound dressing should fulfil many requirements [220], including isolation from harmful external factors, such as secondary wound infection, but should also provide sufficient water vapour permeability and oxygen availability. HGs based on tragacanth gum (TG), sodium alginate (SA) and PVA meet these properties and have potential applications [220]. In addition to good permeability for water vapour and oxygen, these HGs also provide barriers against secondary wound infection. These properties were confirmed by in vitro studies. Additionally, the HGs had haemolytic and mucoadhesive properties. Dressings based on these HGs were able to release antimicrobial active substances (moxifloxacin, an antibiotic from the fluoroquinolone group used to treat a wide spectrum of microorganisms). The results showed prolonged release of up to 24 h without an initial burst release.

**Figure 9:**
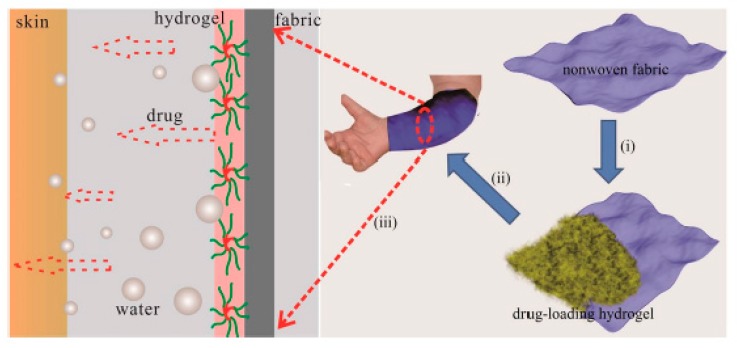
Diagram of a dual-functional fabric; (i) representation showing how the fabric is covered with HG, (ii) representation showing how the HG-coated fabric is applied to the patient’s skin, and (iii) representation showing how the drug diffuses across the skin. Reprinted with permission from Ref. [221].

Patients struggling with atopic skin changes very often use strong steroid drugs to alleviate emerging inflammation. Unfortunately, topical steroid therapy also leads to skin dryness and irritation, which patients with atopic dermatitis should avoid. Therefore, adequate skin hydration in atopic dermatitis therapy is maintained through the systematic and frequent use of appropriate emollients. Wang et al. combined the administration of an anti-inflammatory substance and maintenance of proper skin hydration (Figure 9) [221].

A HG prepared from a mixture of poloxamer 407 (P407) and sodium carboxymethyl cellulose (CMC) was used to achieve this goal (Table 3) [221]. A special nonwoven fabric was covered with the HG mixture. The addition of CMCs resulted in an increase in hydrophilicity of the resulting gel structure and also significantly reduced the sol-gel transition temperature, which advantageously promoted the fabrication of the coated nonwoven fabric. The HG exhibited moisture retaining properties, and the nonwoven fabric material prevented excessive water transpiration. The anti-inflammatory effect was provided by the addition of a Cortex Moutan extract, a well-known plant popular in Chinese medicine, during HG preparation. To determine the drug release profile, in vitro and ex vivo studies were performed. The in vitro studies showed that the release of the anti-inflammatory substance occurred in a prolonged manner up to several dozen hours, ensuring prolonged and elevated levels of the anti-inflammatory substance. Ex vivo tests on pork ear confirmed the results of the in vitro tests. The nonwoven fabric coated with a mixture of HGs composed of P407 and CMCs and Cortex Moutan extract showed potential for treating and caring for atopic lesions in patients with active skin inflammation.

### 5.3. Hydrogels for Ocular Applications

The administration of a slightly soluble substance in the eye can be quite challenging [12,222]. Chitosan-based HGs showed promise as polymeric carriers for both hydrophilic and lyophilic drugs for ocular applications [234]. HGs based on carboxymethyl chitosan (CMCTS) and polyaldehyde dextran (PAD) were prepared [12]. The poor water solubility of the active substance (voriconazole), was addressed by encapsulating in cyclodextrin, which significantly increased the bioavailability of voriconazole and facilitated its application. The HG has the ability to gel in situ, allowing for easier intraocular injection. However, the prolonged release of voriconazole from the gel remained an issue.

Scientists from the Ocean University of China focused on the gelling process [222]. HGs based on hydroxyethylated chitosan (HECTS) with a special azide group showed the ability to polymerize under UV radiation. The polymerization lasted up to 30 s and was carried out in vivo. While the experiments, which were performed on New Zealand rabbits, should be confirmed on the human body, they already gave promising results and were thriving to recognize this HG as a good carrier for the intraocular administration of anti-glaucoma medications.

### 5.4. Hydrogels for Vaginal Applications

A HG made of P407 with nanosized layered double hydroxides allowed to include both hydrophilic and hydrophobic substances in the gel structure, which significantly widens the spectrum of administered drugs to patients [2]. Additionally, the HG has an important ability to gel transition at body temperature, so that during application it is a solution and in contact with the human body temperature it solidifies and stays on the tissues. As sample drugs, water-soluble theaflavin and Nile red dye were used to determine release profiles from the HG in rabbit vagina in independent and simultaneous states [2]. The antiviral properties of theaflavin were preserved and was able to limit the entry of HIV into the immune system. These tests were repeated against nifeviroc as a hydrophobic active and the effect was amplified. Thus, this HG acts as a potential carrier for the delivery of a broad spectrum of antiviral drugs to the human body.

HGs have also been applied in the vaginal administration of antiviral drug in the treatment of HIV [174]. HGs based on CTS with glycerol phosphate gelled upon contact with the human body, at a temperature of approximately 37 °C. Additionally, by enclosing active substances (e.g., tenofovir) in microspheres and immobilizing them in the HG structure (PP gels, Figure 7), a much longer release profile was obtained when compared with the release profile from the microspheres or HG alone. This provides an opportunity to improve on the effectiveness of immunotherapy.

### 5.5. Hydrogels for Topical Oral Applications

In the use of HG structures in the treatment of inflammatory periodontal bent, Chang et al. prepared a gel based on carboxymethyl-hexanoyl chitosan (CHC) [223]. Through the addition of glycerol phosphate into the HG network, the HG was modified to solidify at human body temperatures, which provided significant advantages over the use of other substances in oral inflammation therapy. Anti-inflammatory properties were carried out using naringenin. Experiments on rats showed that the anti-inflammatory substance was more rapidly released from the HG when the pH of the environment was acidic (~5.5), thereby rapidly treating the periodontal inflammation.

CTS HG can also be used to treat oral inflammation when used with thymol [224]. In vitro studies showed that the resulting structure allowed for the prolonged release and high antimicrobial activity of thymol against microorganisms up to 72 h. Furthermore, in the first two days, the active substance was increasingly released. This dual action HG demonstrated anti-inflammatory properties and the treatment of bacterial biofilms in the oral cavity.

### 5.6. Injection of Hydrogels

Therapeutic injections are usually least favoured by the patient and requires specialized HGs. The key challenge is to prepare a structure that will be fluid enough to squeeze through the needle but also sufficiently rigid that when applied does not spread and remains at the injection site [17,135,136]. Therefore, an important property of these structures is shear thinning, i.e., the ability to decrease viscosity of fluid under shear strain.

CTS-based in situ HGs are frequently adopted in the treatment for age-related macular degeneration (AMD), glaucoma and mucosal allergic diseases [234]. The HGs offer a convenient matrix for in situ gelling systems containing other nanoparticles, such as micelles. Injectable CTS-based in situ gels have been applied as in situ forming implants and as drug carriers in nasal and ocular delivery due to good biocompatibility, simple manufacture and sustained-release properties. These systems have been utilized to deliver several chemotherapeutic agents, including camptothecin [218,235], paclitaxel (PTX) [225,226], DOX [226], curcumin [217,227,228], and docetaxel (DTX) [229,236].

An injectable drug delivery system based on a visible light-cured glycol chitosan (GCS) HG containing PTX-complexed beta-cyclodextrin (β-CD) (GCS/CD/PTX) was tested for ovarian cancer therapy using a tumour-bearing mouse model [225]. The swollen GCS HG affected the release of PTX and CD/PTX. These systems exhibited a controlled release of PTX for 7 days. Additionally, GC/CD/PTX resulted in a superior antitumor effect against ovarian cancer.

An interesting application of HGs as dual carriers for PTX and DOX was proposed by Rezazadeh et al. [226]. An injectable thermosensitive HG for simultaneous intra-tumoral administration of PTX and DOX was prepared using mixed micelles composed of Pluronic F127 and α-tocopheryl polyethylene glycol 1000 succinate (TPGS) and a thermosensitive Pluronic F127/hyaluronic acid (PF127/HA) HG containing fixed amount of DOX [226]. PP gel formation temperature, rheological properties, injectability, degradation rates of the HG, and the release rate of DOX and PTX from the HG were examined. The HG containing PTX-loaded micelles and DOX converted to a semisolid with increasing temperature to 35 °C. DOX and PTX were released from the HG at different rates within 12 h and 3 days, respectively. This novel thermosensitive HG could be used for the co-delivery of PTX and DOX in solid tumours.

A series of injectable in situ-forming chitosan-based HGs were prepared by the chemical cross-linking of CTS and genipin in the presence of different inorganic salts [227]. In situ HG formation, with curcumin as an active substance, was detected after subcutaneous injections in rats. In vitro curcumin release profiles exhibited sustained release properties with an initial burst release with approximately three to six times higher cumulative release than other gel controls.

CTS-based HGs may also show magnetic properties, when magnetic Fe_3_O_4_ and MnFe_2_O_4_ nanoparticles are incorporated into their networks [228]. The Fe_3_O_4_ and MnFe_2_O_4_ on CMCTS HGs indicated a pH-sensitive behaviour; their release performances were investigated by curcumin as a model drug. The effects of applied magnetic fields on drug release for composites containing Fe_3_O_4_ were higher compared with MnFe_2_O_4_. The results showed that HGs containing Fe_3_O_4_ and MnFe_2_O_4_ can be applied for novel drug delivery systems.

DTX is an anticancer drug used for treatment of various solid tumours [229]. But it’s low water solubility and a lack of specification has limited its clinical use. Therefore, new solutions have been explored to use this drug in targeted therapy. A thermosensitive chitosan/β-glycerophosphate (CTS/GP) DTX-loaded HG for intratumoral delivery was studied. The results of an in vitro release study demonstrated that DTX-C/GP was released over a period of 3 weeks. The tumour volume was minimized by the intratumoral injection of DTX-CTS/GP (at 20 mg/kg in H22 tumour-bearing mice). Further, the in vivo pharmacokinetic characteristics of DTX-CTS/GP correlated well with the in vitro release. DTX-CTS/GP significantly prolonged DTX retention, supported a high DTX concentration in tumours, and toxicity was effectively reduced.

Carbon nanostructures were also successfully applied in hydrophilic, porous materials as a component of HGs for DTX carriers [236]. A functionalized graphene oxide (GO)-based thermosensitive HG loaded with DTX for intratumoral delivery was designed. First, GO was functionalized by using chitosan to achieve high stability in physiological solutions. Next, HGs containing few components were formed: GO-chitosan, Poloxamer 407 and Poloxamer 188 and DTX. GO/chitosan HG was stable in physiological solution; DTX released much more slowly from this gel compared with free DTX with a pH-responsive feature. The DTX–GO/chitosan gel released higher concentrations and longer resident times in the tumour tissues of mice in vivo giving nontoxic effects to normal organs. Additionally, the combination of near-infrared laser irradiation at 808 nm significantly enhanced tumour inhibition in vitro and in vivo.

**Figure 10:**
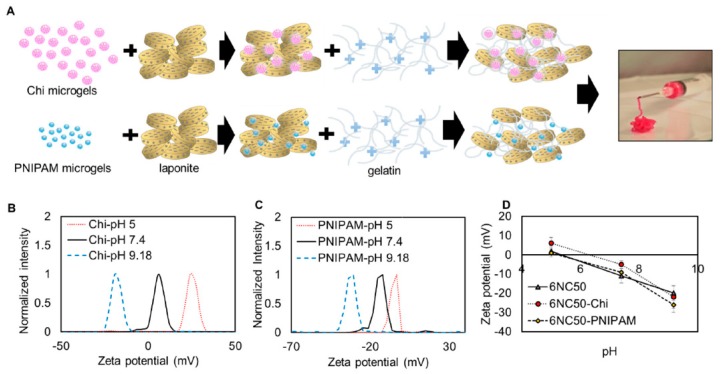
Electrostatic interactions between gelatin and laponite resulted in formation of pH responsive nanocomposite. (**A**) Schematic preparation of shear-thinning HG with laponite. Zeta potential at different pH of (**B**) CTS particles, (**C**) PNIPAM-*co*-Acrylic acid particles and (**D**) PNIPAM-*co*-Acrylic acid and laponite particles. Reprinted with permission from Ref. [230].

Other composite-based HGs were also evaluated. Gharaie et al. used gelatin and laponite in various proportions to obtain appropriate shear thinning properties (Figure 10) [230]. Additionally, by loading with CTS or poly(*N*-isopropylacrylamide)-co-acrylic acid (PNIPAM-co-AA) to the gel structure, pH-responsive abilities were gained, resulting in a different release profiles. As a model substance, rhodamine B was used to test the release. The results of these experiments showed that the gel could be easily and accessibly applied, whereas the release of the active substance was dependent on the pH level (in acidic and neutral environments, the release was negligible, whereas release increased at basic pH.

The key point of interest of many scientists is the fight against cancer. Also in this case HG can also be used to treat cancer [36]. Hu et al. combined salecan, a beta-glucan polysaccharide, with Fe_3_O_4_ nanoparticles and agarose [231]. The resulting HG gained magnetic properties and was used as a carrier for DOX (an anthracycline cytostatic used in anti-cancer therapy). The salecan-based HG with iron oxide nanoparticles had a pH-dependent drug release profile. In an acidic environment, unlike alkaline environments, a larger amount of DOX was released. Due to the acidic pH of cancer cells, this result indicated significant promise. Additionally, due to the presence of Fe_3_O_4_ nanoparticles, the amount of active drug released was also manipulated. With an external electromagnetic field, inorganic particles can be vibrated, thereby loosening the gel structure enabling easier release of DOX.

The technical aspects of DOX release from HGs were examined by a group from Drexel University [232]. In this study, an agarose-based HG was used wherein DOX-dextran sulphate complexes were provided. The structure was doped with divalent metallic ions, Ca^2+^ from CaCl_2_ and Mg^2+^ from MgCl_2_, at different concentrations. The divalent ions interfered with the binding between DOX and dextran sulphate, promoting prolonged release of the active substance from the HG and thereby providing a sustained chemotherapeutic effect for destroying cancer cells. Alginate (sodium alginate, SA)-based HGs with immobilized bevacizumab were also examined [233]. Anti-angiogenic active ingredients were released at prolonged and elevated levels to cancerous and neighbouring cells. As a result, tumour growth was inhibited.

## 6. Future Outlook

Recent developments of HGs as drug delivery carriers for biological and biomedical applications were reviewed. Major synthetic strategies for the preparation of HGs, their classification, the physicochemical properties and their applications were described in detail. The wide variety of recently reported work has demonstrated the application of HG materials as drug delivery systems with different dimensions (macro, micro and nano) in various administration routes.

There are advantages in using HGs as vehicles for active substances. First, HGs can be modified for prolonged or rapid release. This feature is very important in planning therapies for patients and improves compliance with drug regimens. Additionally, by using modified HG materials, drug release can be tailored on demand. Therefore, greater control over health and treatment process can be achieved. Another advantage of functional modifications of HGs is the ability to adjust the controlled release of drugs. By adding specific enzymes or ionisable groups, the entire HG network structure acquires the ability to react to pH changes or to the presence of specific ions.

Technological challenges and problems in the production of HGs are primarily observed in maintaining a balance between chemical structure, composition, drug release and biocompatibility. HGs appear to be very promising materials and forward-looking and have yet to be fully realized. Due to a relatively simple manufacturing process, HGs can be readily modified and functionalized for use in targeted therapy. By using HG structures in medicine, it is easier to achieve better compliance, which is desirable in therapy.

However, the studies reviewed herein are mostly proof-of-concept. There remains limited information on HG in vivo experiments and HG biocompatibility. Most of these studies have focused on in vitro studies to show the non-cytotoxicity of the materials studied. Research on the biocompatibility and biodegradability of these materials, combined with in vivo research, remains a niche topic and has many unresolved questions. The future design and development of effective HG-based drug carriers requires a high degree of control over their properties in vitro and in vivo. These properties include controlled stability for prolonged circulation and biodegradability for facile removal after drug release. One future goal should be the improved design of HG with specific targeting residues to enable highly selective uptake in particular cells or organs. In this way, careful control over size, biodegradability, stability, functionality, and bioconjugation will guide the development of next generation HGs.

## Figures and Tables

**Table 1:** Phase transition temperatures for selected polymers with LCST and UCST.

Polymer Name	Abbreviation	Transition Temperature (in Water)	Refs.
Poly(*N*-isopropylacrylamide)	PNIPAM	30–34 °C	[88]
Poly(*N*,*N*-diethylacrylamide)	PDEAM	32–34 °C	[88]
Poly(methyl vinyl ether)	PMVE	37 °C	[91]
Polyvinyl chloride	PVC	30–50 °C	[92,93]
Gellan gum	-	50–60 °C	[88]
Methylcellulose	-	40 °C	[94,95]
Acrylamide and acrylic acid	AAm and AAc	15–25 °C	[96]

**Table 2:** Examples of HGs with different routes drug immobilization of and their structures and applications.

Type of Loading Drugs	Hydrogel Precursors	Drug	Structure	Applications	Refs.
Post-loading	AA^17^ –BA^18^ –DEAP^19^	17-DMAPG ^15^	Core-shell ^16^	Antitumor activity	[152]
	Carbopol 940 ^20^	Vor ^11^	Core-shell LPN	Dermal applications	[185]
	Carbopol/stearic acid	Vor	Core-shell	Ophthalmic Application	[186]
	PNIPAAm-*b*-PLA-*b*-PEG-*b*-PLA^7^-*b*-PNIPAAm	Riluzole	-	Neuroprotective drug	[183]
	PAA-PNIPAAm	DOX	Core-shell capsule	Antitumor activity	[175]
	PEG-*b*-PDAEMA ^8^	PLGM ^9^	Nanoparticle	Antitumor activity	[172]
	Chitosan/Polysaccharide	Tenofovir	Microsphere	Vaginal drug	[174]
	Chitosan/β-GB	DOX-loaded in LTSL ^29^	PP^33^ HG	Antitumor activity	[187]
	PLGA ^30^ MicroC PEG derivatives (HG)	DOX-loaded in MicroC ^31^ Fu ^32^-loaded in HG	PP HG	Antitumor activity	[188]
In situ loading	Polysaccharide	Ibuprofen	Core-shell capsule	Oral drug	[126]
	Carbohydrate-NIPAM ^22^	Bupivacaine	HG-microgel composite	Anaesthetic drug	[189]
	PNIPAAm-co-AIA ^21^	Lopinavir	Microspheres	Antiretroviral drug	[190]
	CmetCel ^25^ -Dextran	AmB ^23^	MacroHG	Antifungal therapy	[191]
	NiPAAm ^26^-NtBAAm^27^	Fluvastatin	PP HG	HMG-CoA ^25^	[192]
	PPZ ^34^	Silibinin	Microspheres	Anticancer and antiangiogenic activity	[193]
	PEG–PCL ^35^–PEG	PTX micelles ^36^	PP HG	Antitumor activity	[194]

Abbreviations: ^1^ PNIPAAm: poly(*N*-isopropylacrylamide); ^2^ PAA: polyacrylamide; ^3^ PHEMA: poly(hydroxylethyl methacrylate); ^4^ PVP: poly(vinylpyrrolidone); ^5^ PEG: poly(ethylene glycol); ^6^ LPN: lipid nanoparticles; ^7^ PLA: poly(lactide); ^8^ PDAEMA: poly(dehydroabietic ethyl methacrylate); ^9^ PLGM: piperlongumine; ^10^ DOX: Doxorubicin hydrochloride; ^11^ Vor: Voriconazole; ^12^ EGDMA: 2-(2-methyl-acryloyloxy)ethyl 2-methyl-acrylate; ^13^ HEMA: 2-hydroxethyl 2-methylprop-2-enoate; ^14^ Indo: Indomethacin; ^15^ 17-DMAPG: geldanamycin derivative (aminated form, which readily protonates at low pH; ^16^ core-shell structure of HG-in-LPN; ^17^ AA: acrylic acid; ^18^ BA: *N,N’*-methylenebis(acrylamide); ^19^ DEAP: 2,2-diethoxyacetophenone; ^20^ Carbopol 940: HG composed of Precirol ATO 5, Labrafil 1944 CS, and Tween 80; ^21^ AIA: allylamine; ^22^ NIPAM: poly(*N*-isopropylacrylamide); ^23^ AmB: Amphotericin B; ^24^ CmetCel: carboxymethylcellulose-hydrazide; ^25^ HMG-CoA: reductase inhibitor (statin); ^26^ NiPAAm: *N*-isopropylacrylamide; ^27^ NtBAAm: *N*-*tert*-butylacrylamide; ^28^ β-GB: β-glycerophosphate; ^29^ LTSL: lysolipid thermally sensitive liposomes; ^30^ PLGA: poly(lactide-co-glycolide); ^31^ MicroC: microcapsule; ^32^ Fu: 5-fluorouracil; ^33^ PP: “plum padding”; ^34^ PPZ: poly(organophosphazene); ^35^ PCL: poly(ε-caprolactone); ^36^ PTX: Paclitaxel.

**Table 3:** Summary of HGs that can be used as drug carriers.

Hydrogel	Structure/Size	Active Substance	Route of Delivery	Refs.
CTS-g-poly(AA-co-AAm)PVP/MMT	Nanogel	Clarithromycin	Oral	[214]
Salecan/PAMPS	Microgel	Insulin	Oral	[215]
HA	Nanogel	Insulin	Oral	[216]
CTS	Nanogel	Curcumin	Oral	[217]
CTS	-	Camptothecin	Oral	[218]
SS/PVA	Microgel	Gentamycin sulphate	Dermal	[219]
TG/SA/PVA	Microgel	Moxifloxacin	Dermal	[220]
P407/CMCs	Microgel	Cortex Moutan extract	Dermal	[221]
CMCTS/PAD	Macrogel	Voriconazole	Ocular	[12]
HECTS	Macrogel	-	Ocular	[222]
P407	Nanogel	Theaflavin/Nifeviroc	Vaginal	[2]
CTS	Microgel	Tenofovir	Vaginal	[174]
CHC	-	Naringenin	Topical oral	[223]
CTS	Nanogel	Thymol	Topical oral	[224]
GCTS	-	Paclitaxel	Injection	[225]
PF127/HA	Nanogel	Paclitaxel and doxorubicin	Injection	[226]
CTS	-	Curcumin	Injection	[227]
CMCTS	Nanogel	Curcumin	Injection	[228]
CTS/GP	-	Docetaxel	Injection	[229]
Gelatine/laponite (+chitosan or PNIPAM-co-AA)	Nanogel	Rhodamine B	Injection	[230]
Salecan	-	Doxorubicin	Injection	[231]
Agarose	-	Doxorubicin	Injection	[232]
SA	Nanogel	Bevacizumab	Injection	[233]

Abbreviations: CTS: chitosan; AA: acrylic acid; AAm: acrylamide; PVP: polyvinylopyrrolidone; MMT: montmorillonite; PAMPS: poly(2-acryloamido-2-methyl-1-propanesulfonic acid); HA: hyaluronic acid; SS: sericin; PVA: polyvinyl alcohol; TG: tragacanth gum; SA: sodium alginate; P407: poloxamer 407; CMCs: sodium carboxymethyl cellulose; PAD: poly aldehyde dextran; HECTS: hydroxyethylated chitosan; CHC: carboxymethyl-hexanoyl chitosan; GCTS: glycol chitosan; CTS/GP: chitosan/β-glycerophosphate; PNIPAM: poly(*N*-isoproplacrylamide)-co-acrylic acid.
